# Domestic Summary

**Published:** 1840-01-18

**Authors:** 


### DOMESTIC SUMMARY.

*Observations on Dextrine and Diastase.* By William Procter, jr.—Few subjects of the same extent have created more sensation, or have excited more difference of opinion, than *amidon*, and the substances which either constitute it proximately, or are derived from it by chemical agency. Ever since the discovery by M. Raspail, that the grains of fecula were real organs, and not a homogeneous proximate principle, as was before supposed, numerous writers and experimenters have engaged in the question as to what is its chemical constitution? And so various have been the views entertained by many of these, that the task now undertaken, which is to give an outline of the history of the subject, and make such experiments as may assist us in arriving at the truth, so far as it has yet been developed, is one that requires no little industry and perseverance.

As early as December, 1825, M. Raspail announced, in the Annales des Sciences Naturelles,* that fecula of potatoes was composed of two parts, one soluble, and the other insoluble in water. This naturalist stated that the last enveloped the first, which he considered to possess all the characters of gum arabic.

M. Caventou, in the Annales de Physique et de Chemie, tome xxxi, 1825, contradicts the opinion of M. Raspail, and is persuaded that amidon is a proximate vegetable principle, *pure* and *homogeneous.*

M. Guibourt, in the same work, vol. xl, 1829, thought the two parts of amidon differed more in their form than in their chemical nature, and under this relation he regarded them as constituting only one immediate vegetable principle.

* The short historical notices of the several writers following, were obtained from a paper, by M. Guérin Varry, Ann. de Chem. et d'Phys. tome lvi.

In 1829, M. Chevreul, according with the discovery of M. Raspail, ceased to consider amidon as one kind of proximate principle, but he did not admit with him that the soluble matter was *gum*, since it did not possess the property of yielding mucic acid, and he designated the soluble part, under the name of *amidine*, and the insoluble portion by that of *amidin.*

MM. Biot and Persoz, (Nouvelles Annales du Museum, tome ii, 1833,) fully admit with M. Raspail that amidon is composed of two bodies; considering, however, the soluble portion as a pure substance, distinct from *arabine*, to which they gave the name of *dextrine*, by reason that a ray of polarized light deviates strongly to the right in passing through a solid and limpid plate of this substance, or its aqueous solution.

M. Biot states, in a note attached to the above paper, that to prevent all confusion, M. Persoz and himself thought it best to change the name of the soluble matter of fecula, after they had isolated it in sufficient quantities to ascertain all its characters, and to designate it by the property it possessed of turning the planes of polarization to the right, as has been said, stronger than any other organic substance known.

This power or property, in substances, M. Biot called *polarization by rotation*, and M. Fresnel, *circular polarization.*

M. Biot, in his researches on the rotatory power of various vegetable juices, as beet, carrot, radish, etc., ascertained that this property was increased by boiling the juice with the pulp, and he attributes it to the liberation of dextrine from the fecula of the pulp. It is for this reason that M. Biot believes that boiling roots for feeding cattle increases their power of nourishment and renders them more digestible.

MM. Biot and Persoz, in their memoir on the modification of fecula and gums by diluted acids, state that sulphuric acid has the power to rupture the teguments of fecula, and liberate the dextrine, when fecula mixed with water is thrown into a boiling mixture of sulphuric acid and water, and the whole mixture then raised to 196° Fahr. They also say, that by boiling the rotatory power is much diminished. When this solution is filtered and added to a large quantity of alcohol, a white floculent precipitate falls, which is dextrine. When pure dextrine is dissolved in water, and the solution filtered so as to obtain a perfectly limpid solution; and then abandoned to itself, either with or without contact with the air, a white substance gradually precipitates, which has the appearance of inulin. It is not inulin, because, when dissolved in hot water, it turns the planes of polarization to the right, whereas inulin turns it to the left. M. Biot considers this substance a modification of dextrine.

*To be continued.*

